# Transplantation: platform to study recurrence of disease

**DOI:** 10.3389/fimmu.2024.1354101

**Published:** 2024-03-01

**Authors:** George William Burke, Alla Mitrofanova, Antonio Miguel Fontanella, Francesco Vendrame, Gaetano Ciancio, Rodrigo M. Vianna, David Roth, Phillip Ruiz, Carolyn L. Abitbol, Jayanthi Chandar, Sandra Merscher, Alberto Pugliese, Alessia Fornoni

**Affiliations:** ^1^ Division of Kidney-Pancreas Transplantation, Department of Surgery, Miami Transplant Institute, University of Miami Miller School of Medicine, Miami, FL, United States; ^2^ Katz Family Division of Nephrology and Hypertension, Department of Medicine, University of Miami Miller School of Medicine, Miami, FL, United States; ^3^ University of Miami Miller School of Medicine, Miami, FL, United States; ^4^ Division of Endocrinology, Diabetes, and Metabolism, Department of Medicine, University of Miami Miller School of Medicine, Miami, FL, United States; ^5^ Department of Surgery, Miami Transplant Institute, University of Miami Miller School of Medicine, Miami, FL, United States; ^6^ Transplant Pathology, Immunology and Histocompatibility Laboratory University of Miami Department of Surgery, Miami Transplant Institute, University of Miami Miller School of Medicine, Miami, FL, United States; ^7^ Pediatric Nephrology & Hypertension, University of Miami Miller School of Medicine, Miami, FL, United States; ^8^ Pediatric Kidney Transplant, Miami Transplant Institute, University of Miami Miller School of Medicine, Miami, FL, United States; ^9^ Peggy and Harold Katz Family Drug Discovery Center, Department of Medicine, University of Miami - Miller School of Medicine, Miami, FL, United States; ^10^ Department of Diabetes Immunology, Arthur Riggs Diabetes and Metabolism Research Institute, City of Hope, Duarte, CA, United States

**Keywords:** kidney transplant, pancreas transplant, liver transplant, recurrent disease, cytokines

## Abstract

Beyond the direct benefit that a transplanted organ provides to an individual recipient, the study of the transplant process has the potential to create a better understanding of the pathogenesis, etiology, progression and possible therapy for recurrence of disease after transplantation while at the same time providing insight into the original disease. Specific examples of this include: 1) recurrence of focal segmental glomerulosclerosis (FSGS) after kidney transplantation, 2) recurrent autoimmunity after pancreas transplantation, and 3) recurrence of disease after orthotopic liver transplantation (OLT) for cirrhosis related to progressive steatosis secondary to jejuno-ileal bypass (JIB) surgery. Our team has been studying these phenomena and their immunologic underpinnings, and we suggest that expanding the concept to other pathologic processes and/or transplanted organs that harbor the risk for recurrent disease may provide novel insight into the pathogenesis of a host of other disease processes that lead to organ failure.

## 1 Introduction

The field of transplantation of human organs has made enormous strides since the first kidney transplant from a twin donor to his identical twin brother (skin graft proven) was performed by Dr. Joseph Murray, on December 23, 1954, at the Peter Bent Brigham Hospital in Boston, Massachusetts. Since that time remarkable advances have been made, including the next kidney transplant, also by Dr Joseph Murray, the first living twin (but not identical) donor kidney transplant in 1961, which ushered in the era of immunosuppression. Dr Murray was also the first surgeon to perform a deceased donor kidney transplant. In recognition of his pioneering efforts he was awarded the Nobel Prize in physiology and medicine in 1990 (1). Remarkably, nearly 70 years since the first kidney transplant in humans, organs including kidney, liver, pancreas, heart, intestine, lung, and multivisceral are now transplanted successfully in hospitals worldwide. The growth in the availability of transplantation to patients with end stage disease of a variety of organs has been accompanied by an explosion of knowledge in multiple parallel fields including immunology, immunosuppression, physiology, pharmacotherapeutics, and surgical and medical approaches to disease processes. This also provides us with the opportunity to investigate recurrence of disease.

## 2 Recurrent proteinuria after kidney transplantation for focal segmental glomerulosclerosis

Our study of recurrent proteinuria after kidney transplantation for FSGS has led us to identify immunologic pathways, including innate and adaptive immunity, starting with an important role of a sphingolipid-related enzyme. We demonstrated that Sphingomyelin phosphodiesterase acidlike 3b or SMPDL3b, is involved in maintaining the podocyte actin cytoskeleton upon exposure to serum from patients with FSGS, which is felt to contain the putative inciting circulating factor(s). The existence of a circulating permeability factor was first reported by Virginia Savin and her team in 1996 (2). Possible candidates have included soluble urokinase plasminogen activator receptor (suPAR), an IL-6 family cytokine, cardiotropin-like cytokine-1 (CLC-1), and anti-CD40 antibodies (3, 4). Our group has focused our efforts on studying the podocyte, and changes resulting from exposure to such circulating factors, specifically on post-reperfusion kidney transplant biopsies. We noted that rituximab could bind to SMPDL3b on the podocyte membrane surface and prevent or reduce the subsequent development of proteinuria (5). *In vitro* testing demonstrated co-staining of SMPDL3b with synaptopodin, localizing it to the podocyte membrane. The binding of rituximab to SMPDL3b could also be blocked by siRNA to SMPDL3b, confirming the key role of SMPDL3b, since podocytes were shown not to express CD20 (5). In some instances where proteinuria developed despite treatment with rituximab, we evaluated the possible role of CD 80/B7-1, markers of adaptive immunity, first reported by Reiser and Mundel (6). We demonstrated that kidney transplant biopsies performed post-reperfusion in the operating room in those patients who experienced recurrent proteinuria 1) showed foot process (FP) effacement by electron microscopy (EM) (7), and 2) stained positive for podocyte CD 80/B7-1 (8). The pre-reperfusion biopsies showed normal FPs with no effacement and were negative for podocyte B7-1 expression. These patients responded with improvement or resolution of proteinuria after treatment with abatacept (8). This was confirmed in a larger series of patients, underscoring the importance of 1) post-reperfusion biopsies to identify the induced expression of B7-1 or other as yet not identified markers, and 2) treatment with abatacept, which binds to B7-1 with greater affinity than to CD 86/B7-2 and resulted in resolution or improvement in proteinuria in almost all cases (9).

Our team is currently focused on evaluating those cases where recurrent proteinuria develops despite treatment with rituximab and are podocyte B7-1 negative on the post-reperfusion or subsequent biopsies. We are looking for the involvement of other possible innate immune mechanisms, specifically, the cGAS-STING pathway that functions to detect cytosolic DNA presence. We have demonstrated the development of proteinuria after podocyte activation of the cGAS-STING axis (10). This may provide another podocyte – directed focus to treat, and/or prevent recurrence of proteinuria after kidney transplantation for patients with FSGS. Importantly, our work has focused on podocyte – related mechanisms of recurrence, and specifically podocyte-expressed targets (SMPDL3B, B7-1, and cGAS-STING). Utilizing the same platform, we were able to discover a strong and early activation of the TNF pathway in glomerular podocytes from post-reperfusion biopsies of patients transplanted for FSGS (11). Our efforts have underscored the likelihood that recurrent FSGS is associated with numerous inflammatory pathways, some perhaps involving mitochondrial dysfunction (12), as well as innate and/or adaptive immune mechanisms, involving podocytes. Perhaps subgroups exist who may respond to selective intervention, possibly identified in the future by the study of post-reperfusion biopsies in those kidney transplant recipients with recurrence (13).

Future studies should focus on small groups comprised of comprehensively-studied patients with recurrent FSGS (14, 15), potentially shedding light on circulating permeability factors as well as other possible biomarkers. The demonstration of reversal of changes of FSGS after re-transplantation of transplanted kidneys from two patients that experienced severe recurrence into patients with ESRD of other etiologies (16, 17), serves as a striking reminder of the existence of as yet unidentified, circulating factors. We anticipate that the clarification of mechanisms involved in recurrence of FSGS following kidney transplantation will lead to therapies that could then be applied successfully to patients with early FSGS of the native kidneys, thereby preventing progression of injury and possibly avoiding the need for transplantation (13, 14).

## 3 Recurrent autoimmunity after pancreas transplantation

The second area of our investigation of recurrent disease following transplantation involves the study of Type 1 Diabetes (T1D) Recurrence (T1DR) or recurrent auto-immunity, in pancreas or simultaneous pancreas kidney (SPK) transplant recipients. Early work in this field was introduced by the pioneering efforts of Dr. David ER Sutherland who transplanted a portion of living, identical twin donor (tail) of the pancreas, from a non-diabetic twin to their T1D sibling. The T1D siblings experienced early function of their pancreas transplant segments, but all returned to insulin therapy within 8 weeks. Eliminating alloimmunity and rejection from the equation by using an identical twin platform, Dr. Sutherland conclusively demonstrated that T1D was an autoimmune disease, and that autoimmunity could recur in a transplanted pancreas (18). We have seen this occur in about 5% of our patients who received deceased donor organs (not living donor organs), over the past 20 to 30 years (19). Typically, SPK transplant recipients who develop T1DR from recurrent disease have been euglycemic, off insulin, for over 2 to 5 years following SPK transplantation, and then present in diabetic ketoacidosis. Once the acute episode has been treated, most of these patients continue to secrete C-peptide as assessed by mixed meal tolerance test (MMTT). This group of patients has been found to seroconvert for autoantibodies to GAD65, IA-2 and ZnT8 prior to presentation in diabetic ketoacidosis (20), despite receiving maintenance immunosuppression. The seroconversion over time for autoantibodies has proven to be a useful biomarker to predict the development of T1DR (21).

Biopsy of the pancreas transplant for evidence of T1DR usually demonstrates insulitis as well as persistent insulin staining of islet cells, along with infiltration of auto-reactive CD3 positive T cells. In those instances where C-peptide is no longer identifiable in the peripheral blood, ductal cells have been shown to stain for insulin (22), suggesting the presence of circulating factors inducing the intra-cellular synthesis of insulin, despite the inability of ductal cells, which, unlike islet cells, don’t have the secretory capacity/machinery, to secrete insulin.

This same population of T cells as found in the pancreas transplant biopsies, has been identified in the peripheral blood, as well as in the peri-pancreas transplant lymph nodes (20, 21, 23). We have attempted to treat these patients with a number of agents that have been used effectively in human autoimmune diseases. First, ustekinumab, a fully human immunoglobulin monoclonal antibody that blocks the P40 subunit of IL-12 and IL-23, and was approved for Crohn’s disease therapy in 2016 (24). Then low dose thymoglobulin (25) which was used in a clinical trial of new onset T1D patients. And most recently we used a course of risankizumab, a humanized monoclonal antibody directed against the subunit P19 of Interleukin 23, which is approved for the treatment of moderate to severe psoriasis (26). We did not observe significant clinical benefit from any of these three agents. During this time we also treated a T1DR recipient with a three month course of alefacept (27), an anti-CD2 monoclonal antibody and observed an improvement in hemoglobin A1c, reduced insulin requirements and a significant fall in CD4 positive auto-antigen reactive T cells in the peripheral blood (28). Alefacept was taken off the market so our plan of treating this patient with a second three month course of alefacept was not possible. During the same time, a U.S trial (the T1DAL study) using alefacept in individuals with newly diagnosed T1D was initiated by Mark Rigby (27). This study demonstrated encouraging results, but was also stopped due to the lack of availability of alefacept. Nonetheless, this topic remains relevant, since a recent report (29) evaluating islet antigen reactive (IAR) CD4 T cells in peripheral blood mononuclear cell samples from the T1DAL study demonstrated a higher frequency of effector memory, IAR CD4 T cells in the alefacept group. These authors suggested 1) using IAR CD4 T cells as a biomarker, and 2) that the possibility of targeting the CD2 pathway warrants investigation for future therapy. This potentially re-opens the door to evaluating agents that deplete effector memory, CD2+ T cells like Siplizumab, a humanized anti-CD2 monoclonal antibody (30), which could be used in our patients with T1DR. Siplizumab depletes effector memory T cells, decreases T cell activity, inhibits T cell proliferation and enriches naïve and mature regulatory T cells (30, 31). In *in vitro* assays, Siplizumab outperformed both Alemtuzumab and rabbit Anti-Thymocyte Globulin (31). In addition, Siplizumab is currently undergoing a clinical trial (STRIDE) in the US in patients with new onset T1D (32).

We are currently pursuing the possibility of targeting the glucose transporter GLUT1 to selectively block autoreactive T cells in T1DR (33). We continue to look for agents that might interfere with the reactivation of this autoimmune process (28) in our search to apply the study of pancreas transplantation to the immunology of recurrent disease.

We have been contributing our experiences with transplant recipients who develop T1DR by sharing biopsies and blood samples with investigators at the Network for Pancreas Organ Donors with Diabetes (nPOD) (34). nPOD is a world-wide organization of clinicians/diabetologists and basic scientists who share the vision of George Eisenbarth MD which was to optimize the preservation of human pancreases for study by obtaining them from brain-dead organ donors with T1D who were donating their other organs for transplantation. Dr Eisenbarth started nPOD in Colorado in 2010. Our group is nPOD-T, or nPOD-Transplantation, for our T1DR contributions. nPOD has the world’s largest biorepository of human pancreata and associated immune organs from patients with T1D, other forms of diabetes and T1DR. nPOD as an organization recovers, processes, analyzes and distributes high quality biospecimens to researchers around the world. nPOD recently published a genomic data archive to promote “the study of novel genotype:phenotype associations, aiding in the mission of nPOD to enhance understanding of diabetes pathogenesis” and to promote the development of novel therapies (35). We hope that our T1DR contributions will help to achieve the goal of new, effective therapies for T1D.

## 4 Recurrent steatosis after orthotopic liver transplantation for jejuno-ileal bypass-related cirrhosis

The third area of recurrent disease study involves recurrence of steatosis/nonspecific hepatitis in an orthotopic liver transplant (OLT) recipient who underwent OLT for cirrhosis secondary to Jejuno-Ileal bypass (JIB) surgery performed for weight loss in the 1960s, an operation now abandoned. The OLT surgery was performed first with the intention of reversing the JIB at a second operation, in order to avoid enteric content spillage during the transplant procedure. After the OLT, the recipient experienced biopsy-proven recurrent steatosis and hepatitis, as well as several episodes of rejection. Three months after OLT, the patient was taken to the operating room for reversal of the J-I bypass. During that operation, samples were drawn from the mesenteric veins of the proximal and distal bypassed segment (jejunum and ileum), as well as the continuous limb of jejunum. Venous samples from the most proximal aspect of the bypassed segment where greater intraluminal stasis, and higher concentrations of bacterial overgrowth might be expected, demonstrated high levels of cytokines, specifically: IL 1 Beta, IFN gamma, and TNF alpha (36). From an immunologic standpoint, these cytokines may have contributed to the development of steatosis particularly IL-1 Beta and TNF-alpha, which have been identified to be significantly associated with increased risk of Non-Alcoholic Fatty Liver Disease (NAFLD) (37). The high levels if IFN-gamma, while likely not contributing to the development of steatosis (37), is likely to have contributed to the rejection episodes (38) seen on liver transplant biopsies in this case. Reversal of the J-I bypass ultimately resulted in the resolution of both steatosis and rejection with good patient outcome. This case report demonstrates the important role of pro-inflammatory cytokines in the mesenteric venous tree, and their potential impact on the downstream organ: the liver. Presumably, restoration of intraluminal intestinal flow after reversal of the G.I. bypass lead to normalization of venous return to the liver, allowing improvement in liver function and reversal of fatty changes (steatosis) (39), as well as rejection in the liver transplant. This remains an important concept impacting the consideration of the use of donor livers with certain degrees of steatosis for liver transplantation. It appears that a certain amount of steatosis of the donor liver may be reversible when receiving mesenteric venous inflow that does not contain high levels of inflammatory mediators (40). Addressing mediators of inflammation and oxidative stress, as well as pro-inflammatory cytokines, such as IL1-beta and TNF-alpha identified in our study (36), are also significant issues for weight loss protocols involving either bariatric surgery or recently introduced weight loss medications, including GLP-1 agonists and SGLT2 antagonists (41, 42).

## 5 Discussion

In all of these lines of investigation, the platform of organ transplantation has presented an opportunity allowing insight into the immunologic pathways of the pathogenic process of the underlying disease. With suitable numbers of patients, along with tissue and blood specimens (43), the recapitulation of the fundamental aspects of the native disease or pathologic mechanisms can be demonstrated, potentially shedding light into the pathogenesis and possible therapies.

We suggest that the development of recurrent disease after organ transplantation offers a unique reverse translational platform that can be used for promoting interdisciplinary team science approaches to study and understand pathways of disease. This proves once more that the interaction between bedside-to-bench and back again is essential to promote clinically relevant discoveries/research. Furthermore, extension of these concepts into the study of the immunology of recurrence of disease that might occur following the transplantation of other organs is an opportunity that could potentially illuminate the pathogenesis of other native organ injuries that progress to end stage disease requiring transplantation. Such critical insight might lead to effective therapy for native disease so that organ transplantation would not be necessary.

## Data availability statement

The original contributions presented in the study are included in the article/supplementary material. Further inquiries can be directed to the corresponding author.

## Ethics statement

The studies involving humans were approved by University of Miami Institutional Review Board. The studies were conducted in accordance with the local legislation and institutional requirements. Written informed consent for participation in this study was provided by the participants’ legal guardians/next of kin. The animal study was approved by University of Miami Institutional Review Board. The study was conducted in accordance with the local legislation and institutional requirements.

## Author contributions

GB: Writing – original draft, Writing – review & editing. AM: Investigation, Writing – review & editing. AnF: Investigation, Writing – review & editing. FV: Investigation, Writing – review & editing. GC: Investigation, Writing – review & editing. RV: Investigation, Writing – review & editing. DR: Conceptualization, Investigation, Writing – review & editing. PR: Investigation, Writing – review & editing. CA: Investigation, Writing – review & editing. JC: Investigation, Writing – review & editing. SM: Investigation, Writing – review & editing. AP: Investigation, Writing – review & editing. AlF: Conceptualization, Investigation, Writing – review & editing.
